# Case of Būnŏsŭs, Apprehended to Be Spurious

**Published:** 1805-05-01

**Authors:** 


### *Case of Būnŏsŭs, apprehended to be spurious.*

ON December 15, 1804, a female infant, about eight weeks old, was inoculated in the upper part of the right arm, by a gentleman of the faculty, with a lancet armed with bunoserous pus. To prevent the blood escaping, and the effect of the inoculation being thereby lost, her father pressed his finger for a time upon the wound; but not without drawing away from it a considerable spread of blood upon his finger: whence he apprehended, that the pus might also have escaped, and the communication of the disorder not have been effected.

By the eighth day, Saturday, the 22d, a pustule, of nearly the circumference of a seven-shilling piece, had formed at the place of incision, of a yellowish hue, with a dark dent in the middle, the whole surrounded with an areola of inflammation, about the size of a half-crown piece, which, in a day or two more, was rather extended than contracted. In a very few succeeding days, the dark dent assumed a greenish colour; and, in a night or two after, was lost, by coming away with a considerable discharge of pus upon the sleeve. The yellow cast of the pustule, as it dried, turned of the colour of gum, or resin; and, in the centre, a deep hole sufficient for a pin's head continued, whence the discharge first came, and again in a few succeeding nights.

In progress the pustule continued, taking rather a darker shade of brown, and cracked into a furrow right across, with another, meeting it about the middle, at right angles. It also continued rising from the arm; and in the night of the 11th of January, the crust separated from the skin, and no more appeared. The pustule being in its appearance so like to the effect of a former bunosism in the same family, which was proved genuine, the sentiments of Mr. Ring, Dr. Walker, &c. are requested upon it, by

Yours,

*Jan.* 14, 1805. EOSACO.

